# Altered muscle recruitment patterns during isometric shoulder abduction in individuals with chronic upper trapezius pain: a cross sectional study

**DOI:** 10.1186/s12891-022-06030-0

**Published:** 2022-12-27

**Authors:** Hyun-A Kim, Oh-Yun Kwon, Chung-Hwi Yi, Hye-Seon Jeon, Woochol Joseph Choi, Jong-Hyuck Weon

**Affiliations:** 1 Department of Physical therapy, Nagoya University, 1-1-20 Daiko-Minami, Higashi-ku, Aichi Prefecture Nagoya City, Japan; 2 Department of Physical Therapy, College of Health Science, Laboratory of Kinetic Ergocise Based on Movement Analysis, Yonsei University, 1 Yonseidae-gil, Gangwon-Do 26493 Wonju, South Korea; 3 Department of Physical Therapy, Yonsei University, 1 Yonseidae-gil, Gangwon-Do 26493 Wonju, South Korea; 4 Department of Physical Therapy, Joongbu University, 201 Daehak-ro, Chubu-myeon, Chungcheongnam-do Geumsan-gun, South Korea

**Keywords:** Myofascial trigger point, Upper trapezius pain, Shoulder abduction, Altered recruitment pattern, Low load

## Abstract

**Background:**

Upper trapezius (UT) pain with myofascial trigger points (MTrPs) can affect movement at the glenohumeral joint as well as at the scapulothoracic joint. The investigation of muscle recruitment patterns can discern motor control strategies. The purpose of this study was to compare shoulder muscle recruitment patterns and muscle activity according to various loads between individuals with and without chronic UT pain.

**Methods:**

In this cross-sectional study, twenty-four participants that had UT pain with MTrPs and sex, age, body weight matched 24 controls with no UT pain were recruited. Surface EMG electrodes were attached to the UT, the serratus anterior (SA), the lower trapezius (LT) and the middle deltoid (MD). All participants performed isometric shoulder abduction with a load of 25%, 50%, or 75% of the maximum strength at 60° of shoulder abduction. The EMG activity, the activity ratio (SA/UT, LT/UT, MD/UT), and the relative contribution of each muscle activity were calculated.

**Results:**

MD activity was significantly decreased in the UT pain group compared to that in the control group (*p* < 0.05). The EMG activity ratio of SA/UT (*p* < 0.025) and the relative contribution of SA activity to shoulder abduction (*p* < 0.05) were significantly greater in the UT pain group than in the control group in the 25% loading condition.

**Conclusion:**

The results of present study showed that UT pain with MTrPs may increase the relative contribution of SA activity and decrease MD activity at low loads. Altered recruitment patterns of scapular upward rotators can be altered in the proper scapular position, which results in decreased MD activity. Clinicians should consider altered recruitment patterns when managing UT pain.

**Trial registration:**

Clinical Research Information Service: Clinical Research Information Service (KCT0007370; 08/06/2022).

## Introduction

Upper trapezius (UT) pain is one of the most common types of musculoskeletal pain reported in clinical practice [1]. It was reported that 73% of women and 58% of men had tenderness of the UT among 198 adults with neck/shoulder pain [2]. The UT is one of the scapular upward rotators with the lower trapezius (LT) and the serratus anterior (SA) [3]. When the arm is abducted more than 30 °, these muscles must contract to induce an upward rotation of the scapula as a coupled action to produce pure rotation around the center of motion at the scapulothoracic joint. This action further accompanies shoulder abduction at the glenohumeral joint, and plays an important role in functional stabilization [3–5].

UT pain is usually accompanied by myofascial trigger points (MTrPs) [6]. Multiple studies have investigated the features of MTrPs of the UT, including lower muscle oxygenation and higher lactate concentrations [7], lower performance in tests of isokinetic shoulder lifting strength [8], hyperactivation and increased muscle tension [9–11], lower abduction torque in the middle deltoid (MD) and lower activation in the UT [12]. It was found that shoulder abduction torque was significantly decreased when the upward rotation and elevation of the scapula were restricted in the UT pain with MTrPs group [13]. The presence of UT pain with MTrPs may influence movement and performance not only at the scapulothoracic joint but also at the glenohumeral joint [14]. However, this mechanism by which this occurs, has not been sufficiently investigated.

Altered recruitment patterns are explained by impairment of the modulator elements in the neuromuscular system [15]. Muscle activation and timing are especially essential for proper activation of the force couple and functioning of the shoulder complex [16]. A previous study found altered muscle activation time in individuals with latent MTrPs in the UT, but there were no significantly amplified changes during scapular plane elevation without load [17].

The EMG ratio has been used to assess muscle balance and to discern the motor control strategy, such as synergistic contribution and motor pattern [18, 19]. It may reflect an altered motor program by which muscles are recruited to a greater degree [20]. Many studies have used varying loads to confirm muscle activation patterns [17, 21, 22]. Several studies have investigated the effect of load on shoulder muscle recruitment patterns using 25%, 50%, and 75% of maximal load” [4, 22]. However, altered recruitment activation patterns corresponding to various loads have not been investigated in individuals with chronic UT pain accompanied by MTrPs.

The aim of this study was to compare muscle activities, recruitment patterns, and the relative contribution of muscle activity, such as the scapular upward rotators and the primary muscle during shoulder abduction, according to various loads between individuals with and without UT pain accompanied by MTrPs.

## Methods

### Design and participants

**Fig. 1:**
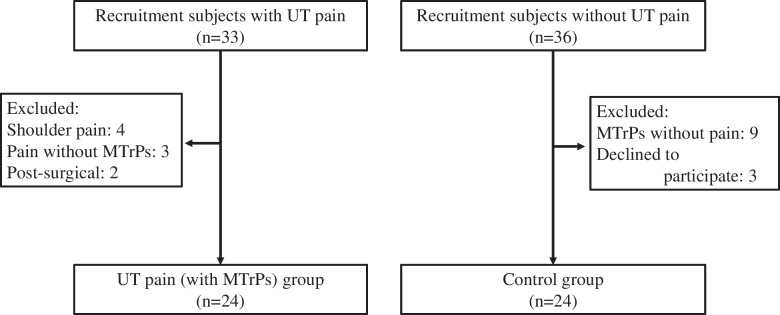
Flow chart showing a summary of participant recruitment

This study is cross - sectional study. The calculation of the sample size was indicated that more than 20 participants were required for each group by G*Power 3.1.9.2 (Franz Faul, Universität Kiel, Germany), using the F tests. First, 69 participants (for the UT pain group: 33, for the control group: 36) were screened by a physiotherapist with more than 5 years of clinical experience to examine MTrPs or lack of MTrPs, using a pressure algometer. In total, 48 participants were recruited (21 participants were excluded) (Fig. 1). The clinical characteristics of the 48 participants are shown in Table 1. The UT pain group included 24 participants who had UT pain with MTrPs (male: 14, female: 10) and the control group included 24 sex-, age-, and weight-matched participants who had no UT pain with MTrPs (male: 14, female: 10). The inclusion criteria for the UT pain group were: (1) sustained and repeated pain in the UT over 3 months, (2) tightness and palpable tender spots in the UT, (3) a self-reported pain visual analog scale (VAS) rating of the UT of > 3 cm, [23] and (4) a pressure pain threshold (PPT) < 2.9 kg/cm^2^ in men and < 2.1 kg/cm^2^ in females [17]. The inclusion criteria for the control group were: (1) no pain in the UT muscle for at least 3 months, (2) no tightness or palpable tender spots in the UT, (3) pain VAS rating of the UT of = 0 cm, and (4) PPT ≥ 2.9 kg/cm^2^ in males, ≥ 2.1 kg/cm^2^ in females [17]. The exclusion criteria were: a previous life-threatening disease, whiplash, trauma, arthritis in the neck or shoulder, or a diagnosis of shoulder impingement syndrome [24]. Before starting the experiment, all participants were informed about the study procedures and provided informed consent to participate. This study was approved by the Yonsei University Wonju Institutional Review Board (1041849-201807-BM-068-02). All the methods were performed in accordance with relevant regulations and guidelines.

**Table 1:** Description of participant characteristics

Characteristics	UT pain^a^ group (*n* = 24)	Control group (*n* = 24)	*p*
Sex (male/female)	m = 14/f = 10	m = 14/f = 10	N/A
Age (years)	24.4 ± 2.7^b^	24 ± 3.2	0.63
Height (cm)	169.6 ± 7.4	169.5 ± 7.6	0.96
Weight (kg)	65.5 ± 11.1	64.2 ± 15.3	0.74
PPT^c^ (kg/cm^2^)	1.6 ± 0.4	3.8 ± 0.8	0.000*
VAS^d^ (mm)	4.7 ± 1.6	N/A	N/A
Pain duration (month)	26.7 ± 30.2	N/A	N/A

^a^*UT pain*: upper trapezius pain

^b^Mean ± standard deviation

^c^*PPT*: Pressure Pain Threshold

^d^*VAS*: Visual Analog Scale

* *p *< 0.05 significant difference.

N/A = not applicable

### Instruments

A Smart KEMA tension sensor (Smart KEMA Measurement System, Factorial Holdings Co., Ltd., Seoul, Korea) was used to measure the maximal isometric strength of shoulder abductors and to control the load (25%, 50%, and 75%) for maximum force. Previous studies have reported high intra-rater reliability of the sensors [13, 25].

**Fig. 2:**
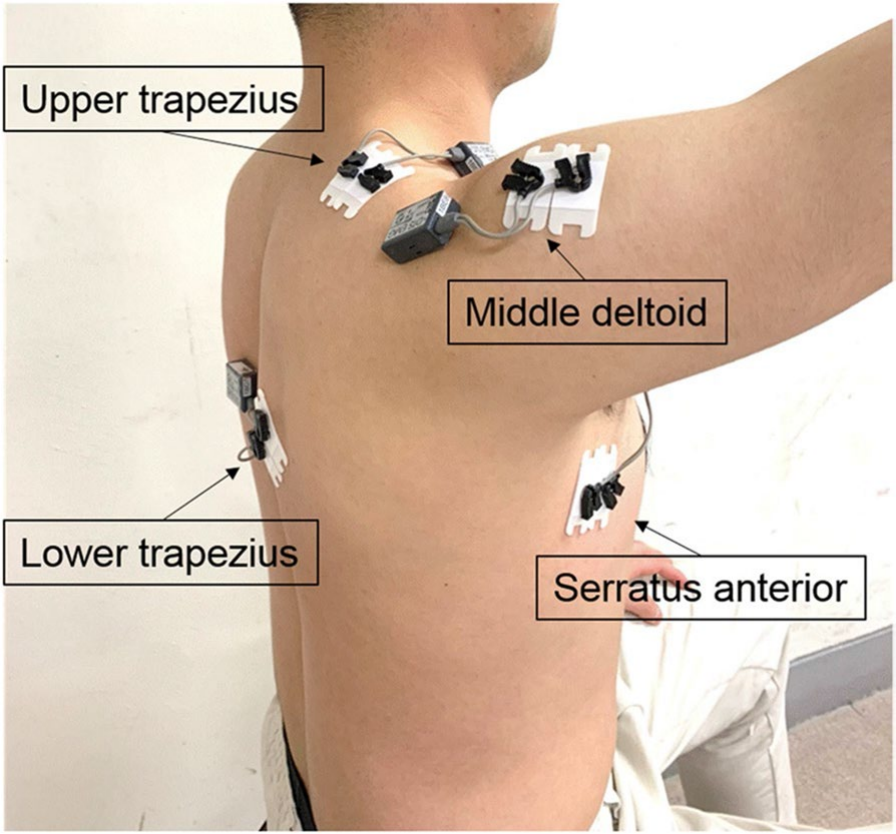
Attachment of surface electromyography electrodes

Surface EMG was used to measure activity during 25%, 50%, and 75% of maximum shoulder abduction isometric strength (TeleMyo 2400T; Noraxon, Scottsdale, AZ, USA). EMG collection software was used at a sampling rate of 1000 Hz. The band-pass filtering was set to 50–450 Hz (Noraxon, Scottsdale, AZ, USA). Surface EMG bipolar Ag/AgCl disposable electrode pairs were attached to the muscle belly of the UT, SA, LT, and MD following the instructions by CRAM (Fig. 2) [26]. For the reduction of surface impedance, the skin was shaved and cleaned with alcohol before the electrodes were attached. EMG measurements during shoulder abduction were performed under three different loading conditions (25%, 50%, and 75% of maximal strength). EMG activity of the UT, SA, LT, and MD was measured during isometric shoulder abduction. The maximum voluntary isometric contractions (MVICs) of each muscle were measured according to Hislop and Montgomery (2007) [27].

### Procedures

**Fig. 3:**
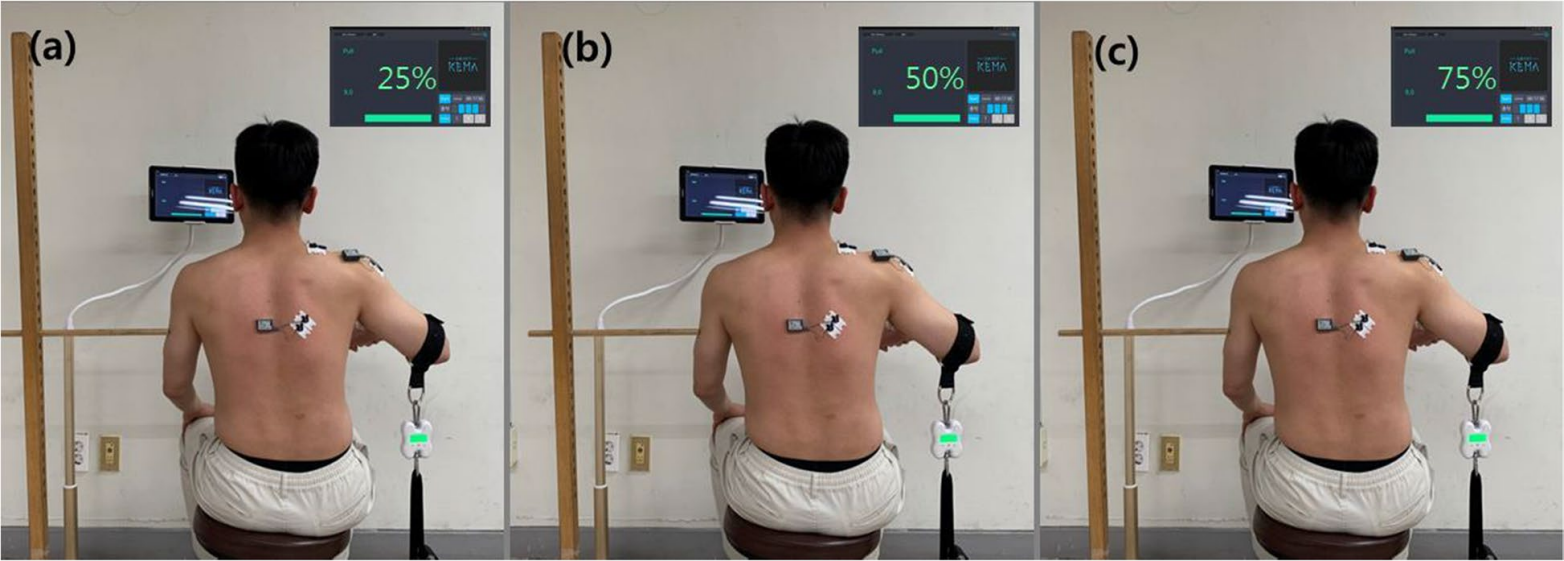
EMG measurements during isometric shoulder abduction by loads. **a** 25% load, (**b**) 50% load, and (**c**) 75% load of maximum strength

EMG was measured during shoulder abduction using different loads (25%, 50%, and 75%). Participants sat on a stool and flexed their elbow 90º with their hands kept in a neutral position. The strap connected to the tension sensor was placed on the distal humerus of the participant. An orthopedic belt connected to the sensor was attached to the grass cup on the floor. The belt was adjusted to 60º of shoulder abduction using a goniometer. Participants were asked to abduct their arm, to pull the tension sensor toward the ceiling with maximum effort for 5 s in the direction of the scapular plane elevation. The three loading conditions of shoulder abduction were determined using a load of 25%, 50%, or 75% of the maximum isometric strength. The participant was asked to practice pulling sensors at 25%, 50%, or 75% of the maximal isometric strength while looking at the screen of a tablet. And they performed pulling the sensor and maintaining isometric strength twice for each condition. (Fig. 3) The allowable margin of error for each resistance was ± 0.5kgf while performing each condition. The tablet was connected to a sensor via Bluetooth and displayed the force shoulder abduction with real-time monitoring during the task. The order of the three conditions was randomized and a random order was determined using www.random.org within each task. The mean values of two measurements were used for all variables. The participants had a rest time for 1 min after each trial and for 2 min after each task for each condition to avoid muscle fatigue.

### Data Analysis

EMG activity was expressed as a percentage of the MVICs for normalization. The MVICs for the UT, SA, LT, and MD were recorded during maximal contraction of each muscle for 5 s twice; the average of the readings from the middle 3 s, for each muscle was used for analysis [28, 29].

The EMG activities (%MVIC) of the SA, LT and MD were each divided by the EMG activity of the UT to obtain activity ratios [19]. The relative contribution of each muscle activity to shoulder abduction performance, was calculated by dividing the activity of each muscle by the sum of the whole activity of all four muscles (using the %MVIC of each muscle) [30].

### Statistic analysis

The Kolmogorov–Smirnov Z-test was used to confirm normal distribution. For the EMG study design, repeated measure analysis of variance (ANOVA) with factors of the groups (participants with UT pain or participants without UT pain) and loads (25%, 50%, or 75% of maximal isometric strength) were used. For all tests, the level of statistical significance was set at *p* < 0.05. For the post-hoc analysis, independent *t*-tests and Bonferroni correction were used to identify specific differences between groups (*α* = 0.025) and loads (*α* = 0.016) when a significant interaction was observed.

## Results

### Muscle activities

Muscle activities of the UT, SA, LT, and MD had significantly different main effects on loads (25%, 50%, and 75%, respectively; *p* < 0.05) (Table 2). EMG activity of the MD was significantly lower in the UT pain group than in the control group (*p* < 0.05) (Table 2). There were no interaction effects in any of the muscle comparisons. Results from the post hoc analysis showed that EMG activity significantly increased with an increase in load in all muscles (*p* < 0.016). In the loading condition of 25%, there was a significant difference only in MD activity between the groups (*p* < 0.025).

**Table 2:** Summary electromyography (EMG) group data. (%MVIC)

	Upper trapezius		Serratus anterior		Lower trapezius		Middle deltoid	
Loads	UT pain^a^	Control	UT pain	Control	UT pain	Control	UT pain	Control
25%	22.6 ± 11.4	24.1 ± 8.0	22.1 ± 7.5	19.2 ± 7.9	20.9 ± 11.2	20.7 ± 11.7	23.1 ± 6.9	33.2 ± 15.9
50%	38.4 ± 15.7	38.5 ± 11.9	33.9 ± 10.9	31.1 ± 13.7	34.1 ± 17.7	33.6 ± 21.3	39.7 ± 12.9	55.8 ± 35.0
75%	57.9 ± 23.6	57.9 ± 19.8	45.3 ± 17.1	42.9 ± 16.3	39.4 ± 21.7	41.1 ± 16.1	56.0 ± 16.5	66.3 ± 27.9
Interaction effect	F = 0.30, *p* = 0.74		F = 0.007, *p* = 0.99		F = 0.347, *p* = 0.71		F = 0.470, *p* = 0.63	
Main effect								
Groups	F = 0.02, *p* = 0.89		F = 0.66, *p* = 0.42		F = 0.01, *p* = 0.93		F = 4.52, *p* = 0.04*	
Loads	F = 89.38, *p* = 0.000*		F = 92.94, *p* = 0.000*		F = 40.79, *p* = 0.000*		F = 148.26, *p* = 0.000*	

^a^*UT pain*: upper trapezius pain group, mean±standard deviation

* *p* <0.05 significant difference

### Motor Control Strategy by the EMG activity ratio

**Fig. 4:**
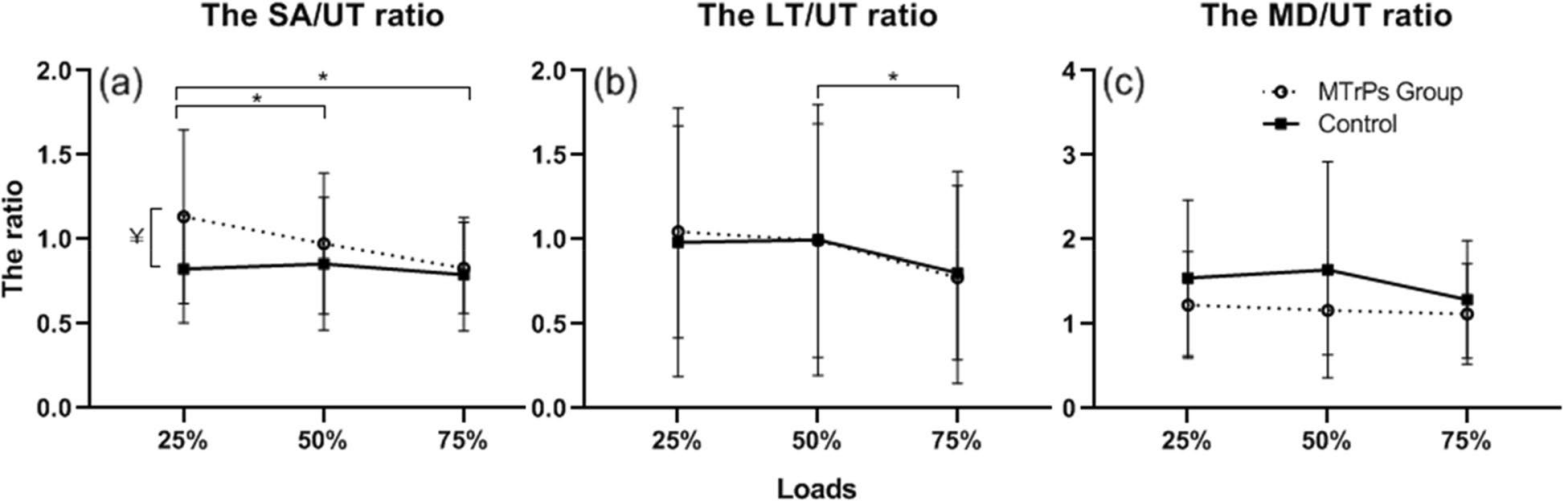
The EMG activity ratio divided by the upper trapezius (UT). SA: serratus anterior, LT: lower trapezius, MD: middle deltoid, MTrPs: myofascial trigger points), * *p* < 0.016 significantly different between the loads, ￥ *p* < 0.025 significantly different between groups

Significant main effects of load (*p* < 0.05) and a significant interaction effect (*p* < 0.05) were seen in case of the EMG activity ration between the SA and UT. Results from the post hoc analysis showed that the EMG activity ratio of SA/UT was significantly greater in the UT pain group than in the control group, only in the loading condition of 25% (*p* < 0.025). The EMG activity ratio of SA/UT was significantly lower in the 50% and 75% loading conditions than in the 25% loading condition (*p* < 0.016, *p* < 0.016) (Fig. 4). There was a significant main effect of load on the EMG activity ratio of LT/UT (*p* < 0.05). Results from the post hoc analysis showed that the EMG activity ratio of LT/UT was significantly lower with a 75% load than with a 50% load (*p* < 0.025). The EMG activity ratio of LT/UT showed no significant main effect of the groups (*p* > 0.05) There was no significant main effect in either load or group, for the EMG activity ratio of MD/UT (p > 0.05).

### The relative contribution of shoulder muscle activity

The main effects between the groups were significantly higher in the SA in the UT pain group than in the control group (*p* < 0.05). In low-load conditions (25%), the relative contribution of SA activity was significantly higher in the UT pain group than in the control group (*p* < 0.025) (Table 3). However, the activity of the UT was significantly higher in the 75% loading condition than in the 25% and 50% loading conditions (*p* < 0.016, *p* < 0.016). The activity of LT was significantly lower in the 75% loading condition as compared to that with a 50% load (*p* < 0.016). There was no significant difference of the SA and MD activities in load (*p* > 0.05).

**Table 3:** Comparison of the relative contribution of shoulder muscle activity between groups according to loads (%)

	Upper trapezius		Serratus anterior		Lower trapezius		Middle deltoid	
Loads	UT pain^a^	Control	UT pain	Control	UT pain	Control	UT pain	Control
25%	25.3 ± 8.6	25.2 ± 7.2	25.0 ± 6.2	19.5 ± 6.0	22.6 ± 9.1	21.5 ± 11.2	27.1 ± 8.5	33.7 ± 12.6
50%	26.1 ± 6.8	25.8 ± 8.2	23.3 ± 6.3	19.7 ± 5.9	22.6 ± 9.2	20.8 ± 8.5	28.1 ± 9.4	33.7 ± 10.8
75%	28.8 ± 6.9	27.9 ± 7.4	22.6 ± 5.6	20.3 ± 5.3	19.4 ± 8.8	19.9 ± 8.0	29.2 ± 9.1	31.8 ± 9.5
Interaction effect	F = 0.16, *p* = 0.85		F = 2.01, *p* = 0.16		F = 2.07, *p* = 0.15		F = 2.09, *p* = 0.15	
Main effect								
Groups	F = 0.04, *p* = 0.85		F = 4.86, *p* = 0.04*		F = 0.12, *p* = 0.73		F = 3.27, *p* = 0.08	
Loads	F = 12.35, *p* = 0.000*		F = 2.31, *p* = 0.12		F = 11.08, *p* = 0.000*		F = 0.27, *p* = 0.77	

^a^UT pain: upper trapezius pain group, mean ± standard deviation

* *p* < 0.05 significant difference

## Discussion

Many previous studies have compared the muscle activities between individuals with and without UT pain [7, 31–33]. However, this is the first reported study to compare muscle activities, the EMG activity ratio, and the relative contribution of primary and synergic muscles during isometric shoulder abduction under different loads (low [25%], middle [50%], and high [75%]) between groups with and without UT pain. The present study investigated the decreased EMG activity of the primary muscle (MD) and altered recruitment patterns of scapular upward rotators in the UT pain group.

This study investigated altered recruitment patterns in the UT pain group. The EMG activity ratio has been used to assess the relative synergistic motor patterns [20]. In the present study, the EMG activity ratio of SA/UT was significantly higher in the UT pain group than in the control group at 25% low-loading condition (UT pain: 1.13, Control: 0.83, *p* < 0.025). In addition, the relative contribution of SA activity in the low-loading condition was significantly greater in the UT pain group than in the control group (UT pain: 25%, control: 19.5%, *p* < 0.025); however, there was no significant difference in the relative contribution of UT activity in this study (UT pain: 25.3%, Control: 25.2%, *p* > 0.05). UT pain with MTrPs seems to lead to a compensatory movement by increasing the relative contribution of SA activity towards force couple motion. Altered recruitment patterns is related to motor element impairments of the nervous system [15].  Changes in recruitment patterns can lead to the action of a synergic muscle to become more dominant than the action of the other synergistic muscles [15]. Clinically it can include consistent recruitment of one muscle of force-couple synergists [15]. The previous study has demonstrated that after the SA was fatigued by electrical stimulation, the muscle activities of other synergies such as the UT and infraspinatus were increased to compensate for SA dysfunction [34]. As seen in previous studies, the findings of the present study postulate that MTrPs can cause functional dysfunction of the UT and can result in altered muscle recruitment patterns of scapular upward rotators. It was investigated that UT activity using surface EMG was lower in the UT pain group than in the control group [12]. When we observed motor control strategy in various loading conditions, the UT pain group showed that the EMG activity ratio of SA/UT was greater under the low-loading condition and decreased with increasing load. In contrast, the control group had a relatively consistent ratio of SA/UT regardless of the loading conditions (Fig. 4). The increased relative activity of the SA in the low loading condition represents a suboptimal motor behavior in motor system adaptation theory, in which activity can be redistributed within and between muscles to protect the tissues from pain involving changes in the motor system [35].

We also observed decreased primary muscle activity in the UT pain group. The MD activity (%MVIC) was significantly decreased in the low loading condition (25%) in the UT pain group compared to the control group (UT pain: 23.1, control: 33.2, *p* < 0.05). This result supports the findings of a previous study that investigated the decreased shoulder abduction force in the UT pain group compared to the control group when scapular elevation and upward rotation were restricted [13]. Altered recruitment patterns of synergistic muscles may cause a movement in the direction of action of the SA muscle. The main standard movement action of the SA is scapular protraction and upward rotation at scapulothoracic joint [3]. Whereas, the standard actions of UT, are not only scapular elevation and upward rotation but also scapular retraction [3]. However when the action of the SA becomes more dominant than that of UT, it can cause the scapular protraction to move toward the glenohumeral joint. This would cause not only decreased dynamic glenohumeral joint stability during shoulder abduction, but also an altered optimal length-tension relationship of the MD muscle. A previous study reported that application of the scapular reposition test, which imparted a force to posteriorly tilt, externally rotate, and retract the scapula improved the length tension relationship of the scapular musculature [36]. As a result, there was reduced pain and increased shoulder elevation strength [36]. An altered scapular position during scapulohumeral movement can change recruitment patterns in scapular stabilizing muscles, which may injure the shoulder joint [37].

It is unclear whether UT pain with MTrPs is a primary phenomenon that may influence people to decrease MD activity, or secondary phenomenon as a result of MD dysfunction. Decreased MD activity can further exacerbate altered recruitment patterns. It was reported that scapular upward rotation during arm elevation was greater in individuals with anterior shoulder tightness than in the control group [38]. Therefore, decreased MD activity in the UT pain group might lead to increased recruitment of the motor unit of the scapular upward rotators through a compensatory movement to induce the same amount of shoulder abduction performance [3, 39].

Most rehabilitation exercises for shoulder impingement syndrome have focused on increasing the EMG activity ratio SA/UT to prevent abnormal patterns of decreased SA and excessive UT activity [19, 40, 41]. However, for UT pain with MTrPs, there seems to be a different motor control strategy pattern compared to that in shoulder impingement syndrome. Our findings showed that UT pain with MTrPs had abnormal patterns with decreased MD and increased relative contribution of SA activity. Previous study postulated that the painful UT might be associated with the lower activity of the UT and decreased shoulder abduction torque [12]. It also highlighted that shoulder abduction and elevation training increased MD strength capacity, UT and MD activation as well as decreased pain in chronic UT muscle [12, 42]. The present study supports the importance of the contractile capacity of the UT and MD under low loads (25% load) during 60° of isometric shoulder abductions.

In this study, all significant differences between groups in EMG activity, EMG activity ratio, and relative contribution were observed only under low loading conditions (25%). It was highlighted that the low-loading condition is useful for managing motor control and that low-load exercise focuses on muscle recruitment patterns and recovery of optimal movement patterns [43]. Therefore, muscle activity tests in low-load conditions might help to determine motor control deficits such as altered muscle recruitment. It was reported that the activity of all muscles increased with load in healthy participants [4]. The present study demonstrated that the UT pain group also significantly increased motor unit firing in all muscles during a graded contraction (25%, 50%, and 75%); this was also seen in the healthy control group.

As shown in Table 3, the relative contribution of the UT increased with an increase in load, but that of the LT decreased with an increase in load. The EMG ratio of LT/UT was also significantly lower at 75% load than at 50% load (*p* < 0.016) (Fig. 4). This means that UT can be overloaded above a 50% load. Previous studies demonstrated that LT activity was significantly increased above 60º in the impingement group. [21] It was noted that LT activity was low at low angles but rapidly increased at high angles [44]. Although LT activity had a tendency for high activity, by increasing load at 60° of shoulder abduction angle in both groups, no significant difference was observed between the UT pain group and the control group in this study. This finding suggests that increased LT activity may have been used as a stabilizer of the scapula rather than a scapular upward rotator as the load increases. However, angle seems to be an important factor in comparing LT activity. Further studies should compare the activity patterns of the LT at angles above 60º to investigate the differences in LT activity between the UT pain and control groups.

This study has several limitations. First, the activity of other scapular stabilizers, such as the rotator cuff muscles, levator scapulae, and rhomboids, were not investigated. The activity of the levator scapulae should be investigated to determine the compensatory movement of scapular elevation with UT. Second, shoulder abduction was performed only at 60° with isometric measurements to avoid the end range of the GH joint. The muscle acitivity patterns can be different during shoulder abductions of over 90°, in the UT pain group. Further studies are needed to determine the effects of low-load exercise to normalize altered recruitment patterns on decreasing UT pain in individuals with UT pain and MTrPs.

## Conclusion

This study compared muscle activities, motor control strategy, and the relative contribution of primary muscle and synergists during 60° of shoulder abduction according to loads (low, middle, high) between the UT pain group and the control group. The results of this study showed that the UT pain group had a significantly increased relative contribution of the SA and the EMG activity ratio of SA/UT. There was a significant decrease in the MD activity under low-loading conditions compared to the control group. Thus, the present study postulates that MTrPs can be the cause of functional dysfunction of the UT muscle and may compensate for UT function by increasing SA activity. Altered recruitment patterns of scapular upward rotators can be altered in the proper scapular position, which results in decreased MD activity. Clinicians should consider low-load exercise focused on normalization of muscle recruitment patterns as an intervention when managing UT pain with MTrPs.

## Data Availability

The datasets used and/or analysed during the current study are available from the corresponding author on reasonable request.

## Abbreviations

UT: Upper trapezius
MTrPs: Myofascial trigger points
SA: Serratus anterior
LT: Lower trapezius
MD: Middle deltoid
MVICs: Maximum voluntary isometric contractions

## References

[1] Ribeiro DC, Belgrave A, Naden A, Fang H, Matthews P, Parshottam S (2018). The prevalence of myofascial trigger points in neck and shoulder-related disorders: a systematic review of the literature. BMC Musculoskelet Disord.

[2] Andersen LL, Hansen K, Mortensen OS, Zebis MK (2011). Prevalence and anatomical location of muscle tenderness in adults with nonspecific neck/shoulder pain. BMC Musculoskelet Disord.

[3] Muscolino JE. The muscular system Manual-E-Book: the skeletal muscles of the human body. Elsevier Health Sciences. 2016. p. 91–108.

[4] Reed D, Cathers I, Halaki M, Ginn KA (2016). Does load influence shoulder muscle recruitment patterns during scapular plane abduction?. J Sci Med Sport.

[5] Page P, Frank C, Lardner R. Assessment and treatment of muscle imbalance: the Janda approach. Human kinetics. 2010. p. 3–9.

[6] De Meulemeester K, Calders P, Dewitte V, Barbe T, Danneels L, Cagnie B (2017). Surface electromyographic activity of the upper trapezius before and after a single dry needling session in female office workers with trapezius Myalgia. Am J Phys Med Rehabil.

[7] Sjøgaard G, Rosendal L, Kristiansen J, Blangsted AK, Skotte J, Larsson B (2010). Muscle oxygenation and glycolysis in females with trapezius myalgia during stress and repetitive work using microdialysis and NIRS. Eur J Appl Physiol.

[8] Hamberg-van Reenen HH, Ariëns GAM, Blatter BM, Twisk JWR, Van Mechelen W, Bongers PM (2006). Physical capacity in relation to low back, neck, or shoulder pain in a working population. Occup Environ Med.

[9] Ge H-Y, Monterde S, Graven-Nielsen T, Arendt-Nielsen L (2014). Latent myofascial trigger points are associated with an increased intramuscular electromyographic activity during synergistic muscle activation. J pain.

[10] Leong H-T, Ng GY, Leung VY, Fu SN (2013). Quantitative estimation of muscle shear elastic modulus of the upper trapezius with supersonic shear imaging during arm positioning. PLoS One.

[11] Westgaard RH, Mork PJ, Lorås HW, Riva R, Lundberg U (2013). Trapezius activity of fibromyalgia patients is enhanced in stressful situations, but is similar to healthy controls in a quiet naturalistic setting: a case-control study. BMC Musculoskelet Disord.

[12] Andersen LL, Nielsen PK, Søgaard K, Andersen CH, Skotte J, Sjøgaard G (2008). Torque–EMG–velocity relationship in female workers with chronic neck muscle pain. J Biomech.

[13] Kim HA, Hwang UJ, Jung SH, Ahn SH, Kim JH, Kwon OY (2017). Comparison of shoulder strength in males with and without myofascial trigger points in the upper trapezius. Clin Biomech.

[14] Bron C, Dommerholt J, Stegenga B, Wensing M, Oostendorp RA. High prevalence of shoulder girdle muscles with myofascial trigger points in patients with shoulder pain. BMC Musculoskelet Disord. 2011;12. http://www.embase.com/search/results?subaction=viewrecord&from=export&id=L51501525%0A10.1186/1471-2474-12-139. 10.1186/1471-2474-12-139 PMC3146907 21711512

[15] Sahrmann S. Diagnosis and treatment of movement impairment syndromes. Elsevier Health Sciences. 2002. p. 9–38.

[16] Camargo PR, Neumann DA (2019). Kinesiologic considerations for targeting activation of scapulothoracic muscles–part 2: trapezius. Brazilian J Phys Ther.

[17] Lucas KR, Rich PA, Polus BI (2010). Muscle activation patterns in the scapular positioning muscles during loaded scapular plane elevation: the effects of latent myofascial trigger points. Clin Biomech.

[18] Arshadi R, Ghasemi GA, Samadi H (2019). Effects of an 8-week selective corrective exercises program on electromyography activity of scapular and neck muscles in persons with upper crossed syndrome: randomized controlled trial. Phys Ther Sport.

[19] Cools AM, Dewitte V, Lanszweert F, Notebaert D, Roets A, Soetens B (2007). Rehabilitation of scapular muscle balance: which exercises to prescribe?. Am J Sports Med.

[20] O’Sullivan PB, Twomey L, Allison GT (1998). Altered abdominal muscle recruitment in patients with chronic back Pain following a specific Exercise intervention. J Orthop Sport Phys Ther.

[21] Ludewig PM, Cook TM (2000). Alterations in shoulder kinematics and associated muscle activity in people with symptoms of shoulder impingement. Phys Ther.

[22] Reed D, Cathers I, Halaki M, Ginn KA (2018). Shoulder muscle activation patterns and levels differ between open and closed-chain abduction. J Sci Med Sport.

[23] Marshall P, Murphy B (2010). Delayed abdominal muscle onsets and self-report measures of pain and disability in chronic low back pain. J Electromyogr Kinesiol.

[24] Andersen LL, Kjær M, Andersen CH, Hansen PB, Zebis MK, Hansen K (2008). Muscle activation during selected strength exercises in women with chronic neck muscle pain. Phys Ther.

[25] Jung S, Hwang U, Kim J, Gwak G-T, Kwon O (2017). Effects of horizontal shoulder abduction and adduction on the activity and strength of the scapular protractors. J Electromyogr Kinesiol.

[26] Cram JR. Introduction to surface electromyography. Aspen Publishers. 1998. p. 289–306.

[27] Hislop HJ (2007). Daniels and Worthingham’s Muscle testing.

[28] Ekstrom RA, Soderberg GL, Donatelli RA (2005). Normalization procedures using maximum voluntary isometric contractions for the serratus anterior and trapezius muscles during surface EMG analysis. J Electromyogr Kinesiol.

[29] Kendall FP, McCreary EK, Provance PG, Rodgers MM, Romani WA (2005). Muscles: testing and function, with posture and pain (Kendall, muscles).

[30] Mapelli A, Machado BCZ, Giglio LD, Sforza C, De Felício CM (2016). Reorganization of muscle activity in patients with chronic temporomandibular disorders. Arch Oral Biol.

[31] Lucas KR, Polus BI, Rich PA (2004). Latent myofascial trigger points: their effects on muscle activation and movement efficiency. J Bodyw Mov Ther.

[32] Vasseljen O, Westgaard RH (1995). A case-control study of trapezius muscle activity in office and manual workers with shoulder and neck pain and symptom-free controls. Int Arch Occup Environ Health.

[33] Ginszt M, Szkutnik J, Zieliński G, Bakalczuk M, Stodółkiewicz M, Litko-Rola M (2022). Cervical Myofascial Pain is Associated with an imbalance of Masticatory muscle activity. Int J Environ Res Public Health.

[34] Umehara J, Nakamura M, Nishishita S, Tanaka H, Kusano K, Ichihashi N (2018). Scapular kinematic alterations during arm elevation with decrease in pectoralis minor stiffness after stretching in healthy individuals. J Shoulder Elb Surg..

[35] Hodges PW (2011). Pain and motor control: from the laboratory to rehabilitation. J Electromyogr Kinesiol.

[36] Tate AR, McClure P, Kareha S, Irwin D (2008). Effect of the scapula reposition test on shoulder impingement symptoms and elevation strength in overhead athletes. J Orthop Sport Phys Ther.

[37] Kibler BW, McMullen J (2003). Scapular dyskinesis and its relation to shoulder pain. JAAOS-Journal Am Acad Orthop Surg.

[38] Lin J, Lim HK, Yang J (2006). Effect of shoulder tightness on glenohumeral translation, scapular kinematics, and scapulohumeral rhythm in subjects with stiff shoulders. J Orthop Res.

[39] Winter DA. Biomechanics and motor control of human movement. Wiley. 2009. p. 276–80.

[40] Chester R, Smith TO, Hooper L, Dixon J (2010). The impact of subacromial impingement syndrome on muscle activity patterns of the shoulder complex: a systematic review of electromyographic studies. BMC Musculoskelet Disord..

[41] Miyasaka J, Arai R, Yoshioka Y, Matsumura A, Hasegawa S, Kuriyama S (2022). Electromyographic analysis of a selective exercise for the serratus anterior muscle among patients with frozen shoulder and subacromial impingement syndrome. Am J Phys Med Rehabil.

[42] Andersen LL, Andersen CH, Zebis MK, Nielsen PK, Søgaard K, Sjogaard G (2008). Effect of physical training on function of chronically painful muscles: a randomized controlled trial. J Appl Physiol..

[43] Aasa B, Berglund L, Michaelson P, Aasa U (2015). Individualized low-load motor control exercises and education versus a high-load lifting exercise and education to improve activity, pain intensity, and physical performance in patients with low back pain: a randomized controlled trial. J Orthop Sport Phys Ther.

[44] Neumann DA. Kinesiology of the musculoskeletal system-e-book: foundations for rehabilitation. Elsevier Health Sciences. 2013. p. 119–67.

